# *N* ^1^-[(1*H*-Imidazol-2-yl)methyl­idene]-*N* ^4^-phenyl­benzene-1,4-di­amine

**DOI:** 10.1107/S1600536814014238

**Published:** 2014-06-25

**Authors:** Md. Serajul Haque Faizi, Ashraf Mashrai, M. Shahid, Musheer Ahmad

**Affiliations:** a Department of Chemistry, Indian Institute of Technology Kanpur, Kanpur, UP 208 016, India; b Department of Chemistry, Aligarh Muslim University, Aligarh 202 002, India

**Keywords:** crystal structure

## Abstract

The title compound, C_16_H_14_N_4_, is non-planar with dihedral angles between the planes of the imidazole and phenyl­enedi­amine rings of 30.66 (4)° and between the planes of the phenyl­enedi­amine and *N*-phenyl rings of 56.63 (7)°. In the crystal, mol­ecules are connected by N—H⋯N hydrogen bonds, generating a chain extending along the *b*-axis direction. The crystal structure is also stabilized by C—H⋯π inter­actions between *N*-phenyl and imidazole rings and slipped π–π stacking inter­actions between imidazole rings [centroid–centroid distance = 3.516 (4) Å] giving an overall two-dimensional layered structure lying parallel to (010).

## Related literature   

For applications of Schiff bases, see: Lozier *et al.* (1975 ▶); Dalapati *et al.* (2011 ▶); Sun *et al.* (2012 ▶). The present work is part of an ongoing structural study of Schiff base–metal complexes, see: Faizi & Hussain (2014 ▶); Faizi & Sen (2014 ▶). For related Schiff bases and their applications, see: Thompson *et al.* (2012 ▶); Shue *et al.* (1994 ▶); Garcia *et al.* (2006 ▶).
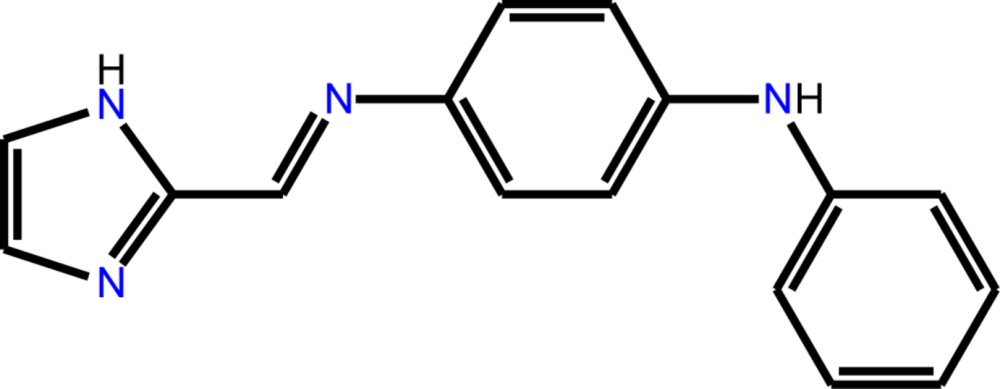


## Experimental   

### 

#### Crystal data  

C_16_H_14_N_4_
*M* *_r_* = 262.31
Monoclinic, 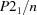

*a* = 15.663 (5) Å
*b* = 5.063 (3) Å
*c* = 16.800 (5) Å
β = 93.124 (5)°
*V* = 1330.3 (10) Å^3^
*Z* = 4
Mo *K*α radiation
μ = 0.08 mm^−1^
*T* = 100 K
0.15 × 0.13 × 0.10 mm

#### Data collection  

Bruker SMART APEX CCD diffractometer
Absorption correction: multi-scan (*SADABS*; Sheldrick, 2004 ▶) *T*_min_ = 0.984, *T*_max_ = 0.990
11186 measured reflections
3296 independent reflections
2403 reflections with *I* > 2σ(*I*)
*R*_int_ = 0.042

#### Refinement  

*R*[*F* ^2^ > 2σ(*F* ^2^)] = 0.044
*wR*(*F* ^2^) = 0.110
*S* = 1.03
3296 reflections
189 parameters
H atoms treated by a mixture of independent and constrained refinement
Δρ_max_ = 0.24 e Å^−3^
Δρ_min_ = −0.18 e Å^−3^

### 

Data collection: *SMART* (Bruker, 2003 ▶); cell refinement: *SAINT* (Bruker, 2003 ▶); data reduction: *SAINT*; program(s) used to solve structure: *SIR97* (Altomare *et al.*, 1999 ▶); program(s) used to refine structure: *SHELXL97* (Sheldrick, 2008 ▶); molecular graphics: *DIAMOND* (Brandenberg & Putz, 2006 ▶); software used to prepare material for publication: *DIAMOND*.

## Supplementary Material

Crystal structure: contains datablock(s) global, I. DOI: 10.1107/S1600536814014238/gg2140sup1.cif

Structure factors: contains datablock(s) I. DOI: 10.1107/S1600536814014238/gg2140Isup2.hkl

Supporting information file. DOI: 10.1107/S1600536814014238/gg2140Isup3.cml

CCDC reference: 1008808

Additional supporting information:  crystallographic information; 3D view; checkCIF report

## Figures and Tables

**Table 1:** Hydrogen-bond geometry (Å, °) *Cg*1 is the centroid of the N3/N4/C14–C16 ring.

*D*—H⋯*A*	*D*—H	H⋯*A*	*D*⋯*A*	*D*—H⋯*A*
N4—H101⋯N3^i^	0.86	2.09	2.875 (3)	151
C2—H2⋯*Cg*1^ii^	0.93	2.83	3.691 (3)	155

Symmetry codes: (i) 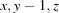; (ii) 
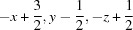
.

**Fig. 1.**
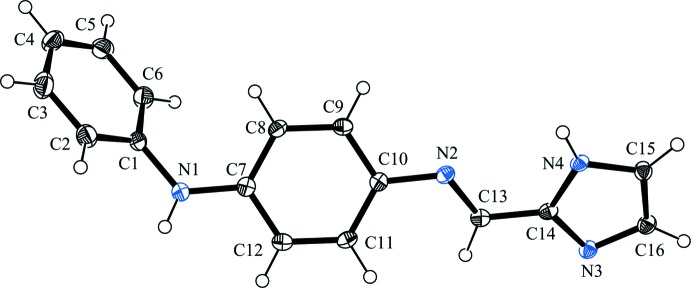
The molecular conformation and atom-numbering scheme for the title compound with non-H atoms drawn as 40% probability displacement ellipsoids.

**Fig. 2.**
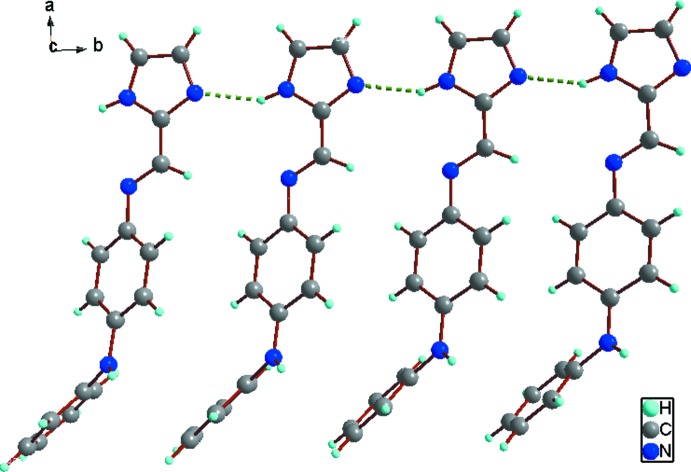
The one-dimensional hydrogen-bonded chain structure in the title compound extending along c, with hydrogen bonds shown as dashed lines.

**Fig. 3.**
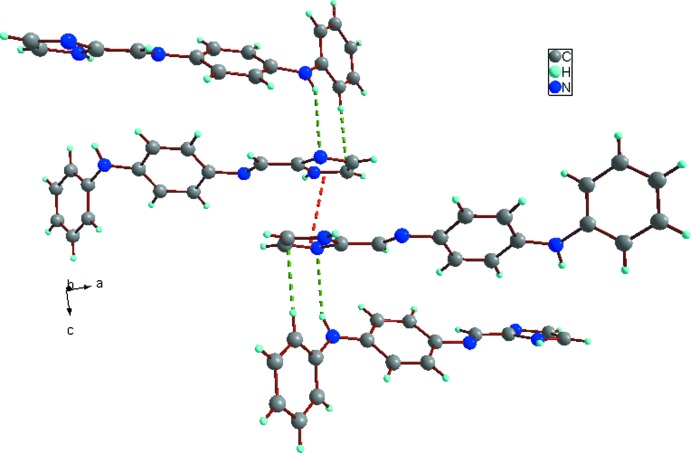
The two-dimensional weak bond interaction present in the title compound extending along b, with weak bond interaction shown as dashed lines.

## supplementary crystallographic information

### S1. Comment

Schiff bases often exhibit various biological activities and in many cases were shown to have antibacterial, anticancer, anti-inflammatory and antitoxic properties (Lozier *et al.*, 1975). They are used as anion sensors (Dalapati *et al.*, 2011) and as non-linear optics compounds (Sun *et al.*, 2012). The present work is part of an ongoing structural study of Schiff base metal complexes (Faizi & Hussain, 2014; Faizi & Sen, 2014) and we report here the structure of N1-((1*H*-imidazol-2-yl)methylene)-N4-phenylbenzene-1,4-diamine (IMPD). There are very few examples similar to title compound and their metal complex have been reported in the literature (Thompson *et al.*, 2012; Shue *et al.*, 1994; Garcia *et al.*, 2006). The synthesis of IMPD by condensation of 2-imidazolecarboxaldehyde and *N*-phenyl-*p*-phenylenediamine has not previously been reported. In the title compound (Fig. 1) IMPD has non planar structure, the dihedral angle between the imidazole and phenylenediamine rings is 30.66 (4) ° and the dihedral angle between the phenylenediamine and *N*-phenyl rings is 56.63 (7) °. The imine group displays a torsional angle (C10—N2—C13—C14) of 177.29 (2)°. In the crystal, molecules are connected by intermolecular N—H···N hydrogen bond interaction generate a one-dimensional chain structure extending along *c* axis (Table 1, Fig 2). The crystal structure is also stabilized by C—H···π interations between *N*-phenyl and imidazole and slipped π–π stacking interactions between imidazole rings [centroid–centroid distance = 3.516 (4) Å] give an overall two-dimensional layered structure lying parallel to (010) given in Fig 3.

### S2. Experimental

100 mg (1 mmol) of *N*-phenyl-*p*-phenylenediamine were dissolved in 10 ml of absolute ethanol. To this solution, 52 mg (1 mmol) of 2-imidazolecarboxaldehyde in 5 ml of absolute ethanol was dropwisely added under stirring. Then, this mixture was stirred for 10 min, two drops of glacial acetic acid were then added and the mixture was further refluxed for 2h. The resulting light green precipitate was recovered by filtration, washed several times with a small portions of EtOH and then with diethyl ether to give 120 mg (86%) of N1-((1*H*-imidazol-2-yl)methylene)-N4-phenylbenzene-1,4-diamine (IMPD). The crystal of the title compound suitable for X-ray analysis was obtained within 3 days by slow evaporation of the MeOH solvent.

### S3. Refinement

All H-atoms were positioned geometrically and refined using a riding model with C—H = 0.92–0.93 Å and *U*_iso_(H) = 1.2*U*_eq_(C).

### Crystal data

**Table:**

C_16_H_14_N_4_	*F*(000) = 552
*M**_r_* = 262.31	*D*_x_ = 1.310 Mg m^−^^3^
Monoclinic, *P*2_1_/*n*	Mo *K*α radiation, λ = 0.71073 Å
Hall symbol: -P 2yn	Cell parameters from 999 reflections
*a* = 15.663 (5) Å	θ = 1.8–25.5°
*b* = 5.063 (3) Å	µ = 0.08 mm^−^^1^
*c* = 16.800 (5) Å	*T* = 100 K
β = 93.124 (5)°	Block, yellow
*V* = 1330.3 (10) Å^3^	0.15 × 0.13 × 0.10 mm
*Z* = 4	

### Data collection

**Table:**

Bruker SMART APEX CCD diffractometer	3296 independent reflections
Radiation source: fine-focus sealed tube	2403 reflections with *I* > 2σ(*I*)
Graphite monochromator	*R*_int_ = 0.042
ω scans	θ_max_ = 28.3°, θ_min_ = 2.4°
Absorption correction: multi-scan (*SADABS*; Sheldrick, 2004)	*h* = −13→20
*T*_min_ = 0.984, *T*_max_ = 0.990	*k* = −6→6
11186 measured reflections	*l* = −22→22

### Refinement

**Table:**

Refinement on *F*^2^	Primary atom site location: structure-invariant direct methods
Least-squares matrix: full	Secondary atom site location: difference Fourier map
*R*[*F*^2^ > 2σ(*F*^2^)] = 0.044	Hydrogen site location: inferred from neighbouring sites
*wR*(*F*^2^) = 0.110	H atoms treated by a mixture of independent and constrained refinement
*S* = 1.03	*w* = 1/[σ^2^(*F*_o_^2^) + (0.0458*P*)^2^ + 0.404*P*] where *P* = (*F*_o_^2^ + 2*F*_c_^2^)/3
3296 reflections	(Δ/σ)_max_ = 0.001
189 parameters	Δρ_max_ = 0.24 e Å^−^^3^
0 restraints	Δρ_min_ = −0.18 e Å^−^^3^

### Special details

**Table:**

Geometry. All esds (except the esd in the dihedral angle between two l.s. planes) are estimated using the full covariance matrix. The cell esds are taken into account individually in the estimation of esds in distances, angles and torsion angles; correlations between esds in cell parameters are only used when they are defined by crystal symmetry. An approximate (isotropic) treatment of cell esds is used for estimating esds involving l.s. planes.
Refinement. Refinement of *F*^2^ against ALL reflections. The weighted *R*-factor *wR* and goodness of fit *S* are based on *F*^2^, conventional *R*-factors *R* are based on *F*, with *F* set to zero for negative *F*^2^. The threshold expression of *F*^2^ > σ(*F*^2^) is used only for calculating *R*-factors(gt) *etc*. and is not relevant to the choice of reflections for refinement. *R*-factors based on *F*^2^ are statistically about twice as large as those based on *F*, and *R*- factors based on ALL data will be even larger.

### Fractional atomic coordinates and isotropic or equivalent isotropic displacement parameters (Å^2^)

**Table:**

	*x*	*y*	*z*	*U*_iso_*/*U*_eq_	
C1	0.43125 (8)	−0.1760 (3)	0.33278 (8)	0.0214 (3)	
C2	0.38397 (9)	−0.3415 (3)	0.28135 (9)	0.0282 (3)	
H2	0.3891	−0.3274	0.2266	0.034*	
C3	0.32914 (10)	−0.5278 (3)	0.31101 (11)	0.0352 (4)	
H3	0.2974	−0.6371	0.2761	0.042*	
C4	0.32145 (10)	−0.5514 (3)	0.39221 (11)	0.0337 (4)	
H4	0.2861	−0.6802	0.4121	0.040*	
C5	0.36660 (10)	−0.3828 (3)	0.44371 (10)	0.0308 (4)	
H5	0.3607	−0.3959	0.4984	0.037*	
C6	0.42048 (9)	−0.1945 (3)	0.41415 (9)	0.0275 (3)	
H6	0.4498	−0.0793	0.4490	0.033*	
C7	0.57160 (9)	0.0512 (3)	0.32757 (8)	0.0199 (3)	
C8	0.61533 (9)	−0.1149 (3)	0.38253 (8)	0.0202 (3)	
H8	0.5862	−0.2506	0.4066	0.024*	
C9	0.70154 (9)	−0.0785 (3)	0.40118 (8)	0.0200 (3)	
H9	0.7299	−0.1937	0.4367	0.024*	
C10	0.74693 (8)	0.1273 (3)	0.36788 (8)	0.0174 (3)	
C11	0.70272 (9)	0.2954 (3)	0.31390 (8)	0.0200 (3)	
H11	0.7316	0.4341	0.2910	0.024*	
C12	0.61719 (9)	0.2582 (3)	0.29428 (8)	0.0217 (3)	
H12	0.5891	0.3725	0.2583	0.026*	
C13	0.87684 (9)	0.3549 (3)	0.38064 (8)	0.0190 (3)	
C14	0.96846 (8)	0.3614 (3)	0.39783 (8)	0.0173 (3)	
C15	1.09852 (9)	0.2263 (3)	0.43150 (8)	0.0199 (3)	
H15	1.1455	0.1208	0.4459	0.024*	
C16	1.09789 (9)	0.4940 (3)	0.42150 (8)	0.0206 (3)	
H16	1.1456	0.6027	0.4281	0.025*	
N1	0.48649 (8)	0.0127 (3)	0.30203 (8)	0.0259 (3)	
N2	0.83589 (7)	0.1392 (2)	0.38775 (6)	0.0185 (3)	
N3	1.01646 (7)	0.5797 (2)	0.40025 (7)	0.0192 (3)	
N4	1.01628 (7)	0.1451 (2)	0.41603 (6)	0.0181 (3)	
H101	0.9981	−0.0150	0.4176	0.022*	
H102	0.4709 (11)	0.072 (4)	0.2555 (11)	0.037 (5)*	
H13	0.8508 (10)	0.525 (3)	0.3660 (9)	0.026 (4)*	

### Atomic displacement parameters (Å^2^)

**Table:**

	*U*^11^	*U*^22^	*U*^33^	*U*^12^	*U*^13^	*U*^23^
C1	0.0150 (7)	0.0220 (7)	0.0272 (7)	0.0025 (5)	0.0010 (5)	0.0033 (6)
C2	0.0250 (8)	0.0332 (9)	0.0260 (8)	−0.0003 (7)	−0.0016 (6)	0.0003 (6)
C3	0.0268 (9)	0.0310 (9)	0.0470 (10)	−0.0053 (7)	−0.0044 (7)	−0.0042 (8)
C4	0.0223 (8)	0.0287 (8)	0.0509 (11)	−0.0015 (7)	0.0083 (7)	0.0113 (7)
C5	0.0254 (8)	0.0362 (9)	0.0316 (8)	0.0038 (7)	0.0071 (6)	0.0098 (7)
C6	0.0242 (8)	0.0320 (9)	0.0262 (8)	−0.0016 (6)	0.0013 (6)	−0.0003 (6)
C7	0.0200 (7)	0.0198 (7)	0.0202 (7)	0.0003 (5)	0.0021 (5)	−0.0021 (5)
C8	0.0208 (7)	0.0172 (7)	0.0230 (7)	−0.0029 (5)	0.0038 (5)	0.0020 (5)
C9	0.0234 (7)	0.0162 (6)	0.0204 (7)	0.0010 (5)	0.0017 (5)	0.0009 (5)
C10	0.0192 (7)	0.0153 (6)	0.0177 (6)	0.0002 (5)	0.0024 (5)	−0.0037 (5)
C11	0.0246 (7)	0.0153 (6)	0.0204 (7)	−0.0026 (5)	0.0040 (5)	0.0005 (5)
C12	0.0254 (8)	0.0183 (7)	0.0213 (7)	0.0011 (6)	0.0015 (5)	0.0028 (6)
C13	0.0228 (7)	0.0165 (7)	0.0178 (6)	0.0015 (6)	0.0027 (5)	−0.0014 (5)
C14	0.0214 (7)	0.0141 (6)	0.0169 (6)	−0.0004 (5)	0.0038 (5)	−0.0007 (5)
C15	0.0180 (7)	0.0182 (7)	0.0234 (7)	−0.0001 (5)	0.0002 (5)	−0.0014 (5)
C16	0.0209 (7)	0.0176 (7)	0.0235 (7)	−0.0028 (5)	0.0020 (5)	−0.0018 (5)
N1	0.0198 (6)	0.0314 (7)	0.0260 (7)	−0.0040 (5)	−0.0027 (5)	0.0092 (6)
N2	0.0208 (6)	0.0169 (6)	0.0179 (6)	−0.0023 (5)	0.0025 (4)	−0.0010 (4)
N3	0.0200 (6)	0.0149 (6)	0.0226 (6)	−0.0015 (4)	0.0018 (5)	−0.0013 (4)
N4	0.0208 (6)	0.0117 (5)	0.0220 (6)	−0.0024 (4)	0.0022 (4)	−0.0001 (4)

### Geometric parameters (Å, º)

**Table:**

C1—C2	1.388 (2)	C9—H9	0.9300
C1—C6	1.390 (2)	C10—C11	1.3990 (19)
C1—N1	1.4060 (19)	C10—N2	1.4163 (18)
C2—C3	1.386 (2)	C11—C12	1.375 (2)
C2—H2	0.9300	C11—H11	0.9300
C3—C4	1.381 (2)	C12—H12	0.9300
C3—H3	0.9300	C13—N2	1.2757 (18)
C4—C5	1.383 (2)	C13—C14	1.449 (2)
C4—H4	0.9300	C13—H13	0.976 (17)
C5—C6	1.383 (2)	C14—N3	1.3358 (18)
C5—H5	0.9300	C14—N4	1.3526 (18)
C6—H6	0.9300	C15—N4	1.3636 (18)
C7—N1	1.3918 (18)	C15—C16	1.366 (2)
C7—C8	1.400 (2)	C15—H15	0.9300
C7—C12	1.402 (2)	C16—N3	1.3757 (18)
C8—C9	1.382 (2)	C16—H16	0.9300
C8—H8	0.9300	N1—H102	0.860 (19)
C9—C10	1.3957 (19)	N4—H101	0.8600
			
C2—C1—C6	118.84 (13)	C9—C10—N2	116.95 (12)
C2—C1—N1	119.97 (14)	C11—C10—N2	124.95 (12)
C6—C1—N1	121.15 (13)	C12—C11—C10	120.90 (13)
C3—C2—C1	120.45 (15)	C12—C11—H11	119.6
C3—C2—H2	119.8	C10—C11—H11	119.5
C1—C2—H2	119.8	C11—C12—C7	121.16 (13)
C4—C3—C2	120.21 (16)	C11—C12—H12	119.4
C4—C3—H3	119.9	C7—C12—H12	119.4
C2—C3—H3	119.9	N2—C13—C14	119.90 (13)
C3—C4—C5	119.69 (15)	N2—C13—H13	124.9 (9)
C3—C4—H4	120.2	C14—C13—H13	115.2 (9)
C5—C4—H4	120.2	N3—C14—N4	111.05 (12)
C4—C5—C6	120.16 (15)	N3—C14—C13	125.08 (12)
C4—C5—H5	119.9	N4—C14—C13	123.84 (12)
C6—C5—H5	119.9	N4—C15—C16	105.97 (13)
C5—C6—C1	120.58 (15)	N4—C15—H15	127.0
C5—C6—H6	119.7	C16—C15—H15	127.0
C1—C6—H6	119.7	C15—C16—N3	110.19 (13)
N1—C7—C8	123.04 (13)	C15—C16—H16	124.9
N1—C7—C12	118.81 (13)	N3—C16—H16	124.9
C8—C7—C12	118.08 (13)	C7—N1—C1	125.46 (13)
C9—C8—C7	120.43 (13)	C7—N1—H102	116.8 (12)
C9—C8—H8	119.8	C1—N1—H102	114.9 (12)
C7—C8—H8	119.8	C13—N2—C10	120.49 (12)
C8—C9—C10	121.43 (13)	C14—N3—C16	105.04 (12)
C8—C9—H9	119.3	C14—N4—C15	107.74 (11)
C10—C9—H9	119.3	C14—N4—H101	126.1
C9—C10—C11	117.97 (13)	C15—N4—H101	126.1
			
C6—C1—C2—C3	−2.1 (2)	C8—C7—C12—C11	1.0 (2)
N1—C1—C2—C3	−179.81 (14)	N2—C13—C14—N3	−172.90 (12)
C1—C2—C3—C4	−0.4 (2)	N2—C13—C14—N4	5.0 (2)
C2—C3—C4—C5	2.1 (2)	N4—C15—C16—N3	0.14 (16)
C3—C4—C5—C6	−1.2 (2)	C8—C7—N1—C1	6.8 (2)
C4—C5—C6—C1	−1.3 (2)	C12—C7—N1—C1	−176.26 (14)
C2—C1—C6—C5	3.0 (2)	C2—C1—N1—C7	−130.27 (16)
N1—C1—C6—C5	−179.36 (14)	C6—C1—N1—C7	52.1 (2)
N1—C7—C8—C9	175.16 (13)	C14—C13—N2—C10	−177.30 (11)
C12—C7—C8—C9	−1.8 (2)	C9—C10—N2—C13	−158.92 (12)
C7—C8—C9—C10	1.8 (2)	C11—C10—N2—C13	25.31 (19)
C8—C9—C10—C11	−0.8 (2)	N4—C14—N3—C16	−0.39 (14)
C8—C9—C10—N2	−176.86 (12)	C13—C14—N3—C16	177.74 (12)
C9—C10—C11—C12	−0.07 (19)	C15—C16—N3—C14	0.15 (15)
N2—C10—C11—C12	175.65 (12)	N3—C14—N4—C15	0.49 (15)
C10—C11—C12—C7	−0.1 (2)	C13—C14—N4—C15	−177.67 (12)
N1—C7—C12—C11	−176.13 (13)	C16—C15—N4—C14	−0.37 (15)

### Hydrogen-bond geometry (Å, º)

Cg1 is the centroid of the N3/N4/C14–C16 ring.

**Table:**

*D*—H···*A*	*D*—H	H···*A*	*D*···*A*	*D*—H···*A*
N4—H101···N3^i^	0.86	2.09	2.875 (3)	151
C2—H2···*Cg*1^ii^	0.93	2.83	3.691 (3)	155

Symmetry codes: (i) *x*, *y*−1, *z*; (ii) −*x*+3/2, *y*−1/2, −*z*+1/2.
